# Scalability assessment of Group-IV mono-chalcogenide based tunnel FET

**DOI:** 10.1038/s41598-018-24209-1

**Published:** 2018-04-16

**Authors:** Madhuchhanda Brahma, Arnab Kabiraj, Dipankar Saha, Santanu Mahapatra

**Affiliations:** 1 Centre for Nano Science and Engineering, Indian Institute of Science, Bangalore, 560012 India; 2 Nano-Scale Device Research Laboratory, Department of Electronic Systems Engineering, Indian Institute of Science, Bangalore, 560012 India

## Abstract

Selection of appropriate channel material is the key to design high performance tunnel field effect transistor (TFET), which promises to outperform the conventional metal oxide semiconductor field effect transistor (MOSFET) in ultra-low energy switching applications. Recently discovered atomically thin GeSe, a group IV mono-chalcogenide, can be a potential candidate owing to its direct electronic band gap and low carrier effective mass. In this work we employ ballistic quantum transport model to assess the intrinsic performance limit of monolayer GeSe-TFET. We first study the electronic band structure by regular and hybrid density functional theory and develop two band *k* · *p* hamiltonian for the material. We find that the complex band wraps itself within the conduction band and valence band edges and thus signifies efficient band to band tunneling mechanism. We then use the *k* · *p* hamiltonian to calculate self-consistent solution of the transport equations within the non-equilibrium Green’s function formalism and the Poisson’s equation based electrostatic potential. Keeping the OFF-current fixed at 10 pA/μm we investigate different static and dynamic performance metrics (ON current, energy and delay) under three different constant-field scaling rules: 40, 30 and 20 nm/V. Our study shows that monolayer GeSe-TFET is scalable till 8 nm while preserving ON/OFF current ratio higher than 10^4^.

## Introduction

Dennard’s scaling theory^1,2^ has acted as a guideline for the semiconductor industry to miniaturize the metal oxide semiconductor (MOS) technology in order to comply with the Moore’s law^3^. According to this theory, the power density remains constant over technology nodes if both the dimension and supply voltage (*V*_D_) are scaled by the same factor (known as constant-field scaling). A *V*_D_ scaling also calls for an equal scaling of the threshold voltage (*V*_T_) to preserve the ON-current (*I*_ON_). Since the subthreshold slope (SS) of a MOSFET does not scale with feature size (rather degrades due to drain-induced-barrier-lowering (DIBL) effect) and limited to minimum value of 60 mV/decade, the OFF-current (*I*_OFF_) increases exponentially with *V*_T_ reduction. It is worth noting that the limited value of SS arises from the thermionic emission of the carriers from the source to the channel in a conventional MOSFET. This OFF-state current leads to significant static power dissipation, which is very crucial for battery operated modern hand-held electronic gadgets (cell phone, tablet etc). As a result, the semiconductor industry has been forced to adopt the energy-inept constant-voltage scaling practice for a decade with a value close to 0.6 V.

Thus next generation transistor which can offer sub-60 mV/decade subthreshold slope is in high demand as it may restore the energy efficient constant-field scaling by enabling the supply voltage reduction below 0.6 V. In this view, some interesting devices like negative-capacitance FETs^4,5^, imapact-ionization FETs^6–8^ and tunnel FETs (TFET)^9–12^ are being explored. Present work is focused on TFET, which is basically a gated p-i-n diode where the carriers are injected from the source to the channel by band-to-band tunneling (BTBT) mechanism^13^ and hence promises to offer very low *I*_OFF_ with steep subthreshold swing. However, poor SS and low ON-current have been observed in Silicon based TFETs^14^ due to large indirect energy band gap (1.12 eV), which imposes inefficient phonon assisted tunneling (PAT)^15^. Recent time has seen an extensive investigation of alternative materials for designing high performance TFET^16–21^. Atomically thin layered materials, also known as 2D materials, have found great significance as TFET channel material due to their planar structure, excellent electrostatic integrity, mechanical flexibility and possibility of having direct band gap with low effective mass. Theoretical analysis has been conducted to estimate the performance of TFET based on different such 2D materials: Graphene nanoribbon^22–24^, transition metal di-chalcogenides (MoS_2_, WS_2_, MoSe_2_, WSe_2_, MoTe_2_ etc)^25,26^, Phosphorene^27–29^ etc. Such study has also been extended to hetero-bilayer^30–32^ and hetero-junction devices^25^. At the same time, experimental devices built upon such 2D materials^33–35^ are also reported.

Lately, a new group of 2D materials, namely group IV mono-chalcogenides (GeSe, GeS, SnSe, SnS etc.), has attracted much attention due to their structural similarities with Phosphorene. Among all of them only GeSe has been reported to have direct band gap and low effective mass^36–42^. Moreover, there are reports of experimental fabrication of GeSe nanostructures and nanodevices^43–49^ and their excellent stability in air^50,51^. GeSe is also available with commercial vendors^52^ for experimental research. However, to our best knowledge there is no study on the assessment of GeSe channel based TFET performance. It motivates us to assess the intrinsic performance limit of GeSe-TFET and compare with other 2D material based TFETs.

In this work, we first conduct density functional theory (DFT) calculation to extract effective mass, band gap and the complex band structure (CBS) of monolayer GeSe. Next we perform transport calculations on GeSe-TFET using self-consistent NEGF-Poisson simulation, the details of which are given in the Methods section. We estimate the figure of merit for static performance namely ON-curent (*I*_ON_) and compare the values with other state of the art 2D materials based TFETs. This is followed by the assessment of dynamic figures of merit namely intrinsic switching delay (*τ*) and the power-delay product (PDP). Our study can be useful to assess the potential of GeSe-TFET at the early stage of technology development.

## Results

In this section we first discuss about the atomic structure of monolayer GeSe that we use for DFT calculations. Then we present the calibration of E-K dispersion generated from our adopted two band *k* · *p* hamiltonian model with the DFT results. This is followed by transport calculations for a double gate TFET.

**Figure 1:**
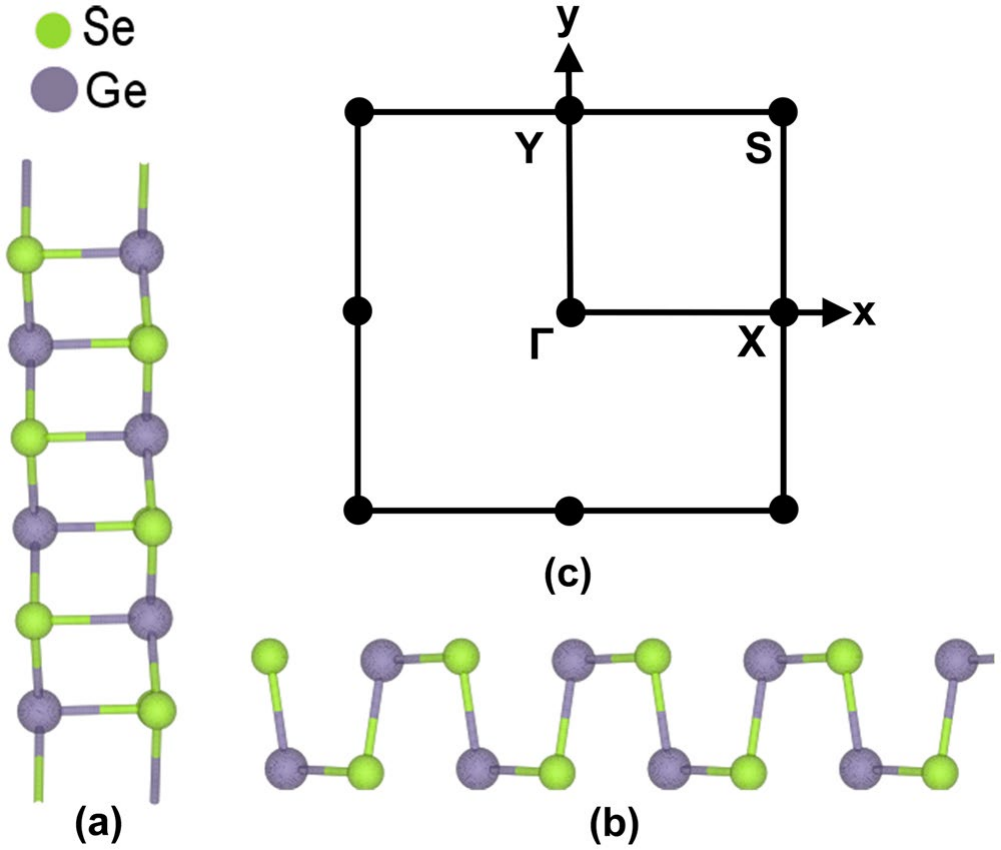
(**a**) Top view and (**b**) Side view of monolayer GeSe (**c**) High-symmetry points in the Brillouin zone.

Figure 1 shows the top and side views of the relaxed atomic configuration of monolayer GeSe including the Brillouin zone. It inherits orthorhombic crystal structure from its bulk counterpart which is a van der Waals layered material. Moreover, monolayer GeSe looks quite similar to the puckered structure of Phosphorene with a slight distortion. It exhibits direct band gap along Γ-X and Γ-Y directions, with a lesser gap along Γ-X direction (See Supplementay Information). The lattice parameters used for electronic structure calculations as well as the values of energy band gap and effective masses obtained from DFT are listed in Table 1. All these values are in agreement with other reported results^40,41^.

**Table 1:** Lattice parameters considered for DFT calculation and resulting electronic properties.

Method		a(Å)	b(Å)	*E*_G_(eV)	${{\boldsymbol{m}}}_{{\boldsymbol{ex}}}^{\ast }$ (*m*_0_)	${{\boldsymbol{m}}}_{{\boldsymbol{ey}}}^{\ast }$ (*m*_0_)	${{\boldsymbol{m}}}_{{\boldsymbol{hx}}}^{\ast }$ (*m*_0_)	${{\boldsymbol{m}}}_{{\boldsymbol{hy}}}^{\ast }$ (*m*_0_)
DFT-PAW	HSE	4.28	3.97	1.6	0.14	0.22	0.15	0.23
	PBE	4.28	3.97	1.15	0.13	0.44	0.14	0.44
DFT-LCAO		4.24	3.99	1.17	0.13	0.57	0.14	0.31

First principles calculations based on two different DFT functionals: Perdew-Burke-Ernzenhof (PBE) exchange-correlation functional^53^ and Heyd-Scuseria-Ernzerhof (HSE) hybrid functional^54^, generate similar band-dispersion with different band gap values. Moreover, the optical band gap value of monolayer GeSe as claimed by commercial vendor^52^ turns out to be 1.6 eV, which has not yet been validated by any scholarly report. Such DFT functional dependent band gap difference is also observed in case of Phosphorene^55–57^. Therefore in our study, we consider it suitable to include DFT calculations based on both methods. Considering both HSE and PBE methods also enable us to study the effect of different band gaps on the scaling behavior of TFETs based on materials with near identical effective mass in the transport direction. We also perform DFT calculations using linear combination of atomic orbitals (DFT-LCAO) in order to compute the complex band structure, which acts as a guideline for material selection.

**Figure 2:**
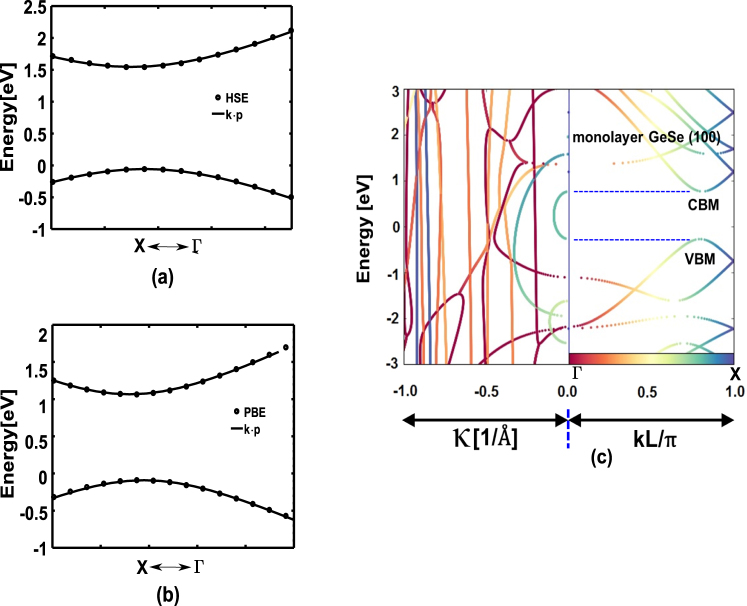
(**a**) DFT-PAW HSE band structure and (**b**) DFT-PAW PBE band structure calibrated with *k* ⋅ *p* model around the band gap regime (see Supplementary Information) (**c**) 2D plot of complex bandstructure of monolayer GeSe (surface cleaved in (100) direction) as obtained from DFT-LCAO. The right-hand panel illustrates the real bands, where the solution **k** are normalized by the perpendicular layer separation **L**. The left-hand panel portrays the complex bands against reciprocal Cartesian coordinates on the x-axis, shows complete wrapping of imaginary branches within CBM and VBM.

In Fig. 2(a) and (b) we show the agreement between the energy dispersion obtained from our calibrated *k* ⋅ *p* model and DFT using projector-augmented-wave (DFT-PAW) method, along the Γ-X direction. From the figure we find that the *k* ⋅ *p* band dispersion matches very well over a range of ≈0.5 eV near conduction band (CB) and valence band (VB) edges, which is sufficient for the tunneling window considered in our transport simulations. We also see in Fig. 2(c) that the imaginary branches of CB and VB completely wrap with each other within the CB mimima (CBM) and VB maxima (VBM), thereby projecting to a very high tunneling probability and insignificant PAT^15^. This allows us to calculate quantum transport under ballistic approximation without considering phonon scattering.

**Figure 3:**
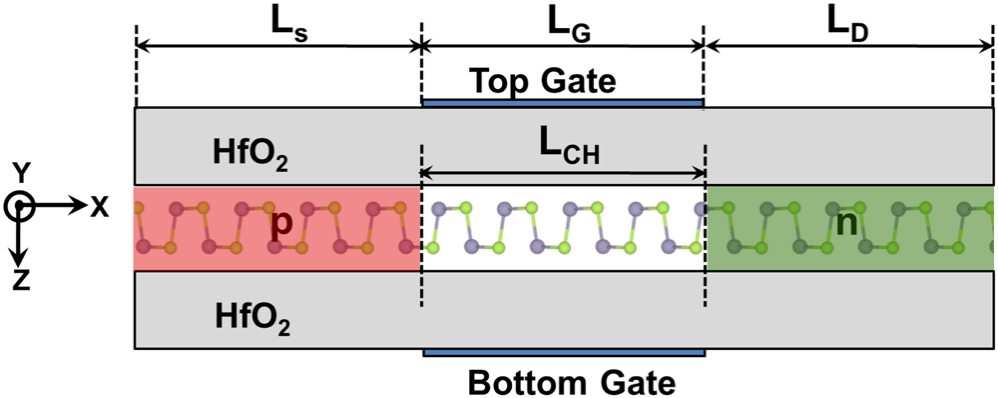
Schematic of the cross-section of the considered GeSe based monolayer TFET. *L*_CH_ denotes the channel length, which equals the gate length *L*_G_. The shaded regions on the left and right represent source (p-doped) and drain (n-doped) respectively.

In our simulation, we have considered a double gate tunnel FET structure as shown in Fig. 3, with effective oxide thickness, EOT = 0.5 nm. The source and drain are uniformly doped p and n regions, while the channel is undoped. The channel length *L*_CH_ is same as the gate length *L*_G_. The source extension *L*_S_ is taken as 16 nm in all the devices under study. The thickness of the GeSe monolayer extracted from the DFT simulations is found to be 0.586 nm. The in-plane and out-of-plane dielectric constants are taken as 13.8^40^ and 1 (ideal 2D case) respectively. The gate work-function is adjusted so that the OFF-state current (*I*_OFF_) at zero gate voltage (*V*_G_) is set at 10 pA/μm for all the devices under study as specified in International Technology Roadmap for Semiconductors (ITRS) for low power nodes^58^. From hereon, all the results obtained from HSE and PBE parameters are marked as HSE and PBE on the plots and in the text.

As pointed out earlier, our main objective is to predict the scaling behavior of GeSe based tunnel FET under the constant-field scaling methodology. Therefore, we have considered three different scaling rules and varied the channel length and *V*_D_ accordingly as shown in Table 2. In doing so, we have to vary the doping concentration as well as the drain length extensions, in order to achieve *I*_ON_ − *I*_OFF_ ratio of 10^4^ and charge neutrality at the contacts respectively (see Supplementary Information).

**Table 2:** Values of ON-current for devices considered under different scaling rules.

*V*_D_		Scaling rule (nm/V)					
		40		30		20	
		HSE	PBE	HSE	PBE	HSE	PBE
0.5 V	*L*_CH_ (nm)	20		15		10	
	*I*_ON_ (μA/μm)	5.05	23.29	1.74	16.30	1.07	6.09
0.4 V	*L*_CH_ (nm)	16		12		8	
	*I*_ON_ (μA/μm)	0.75	8.17	0.62	5.32	0.13	0.22
0.3 V	*L*_CH_ (nm)	12		9		6	
	*I*_ON_ (μA/μm)	0.09	1.37	0.05	0.13	0.0062	0.0061

**Figure 4:**
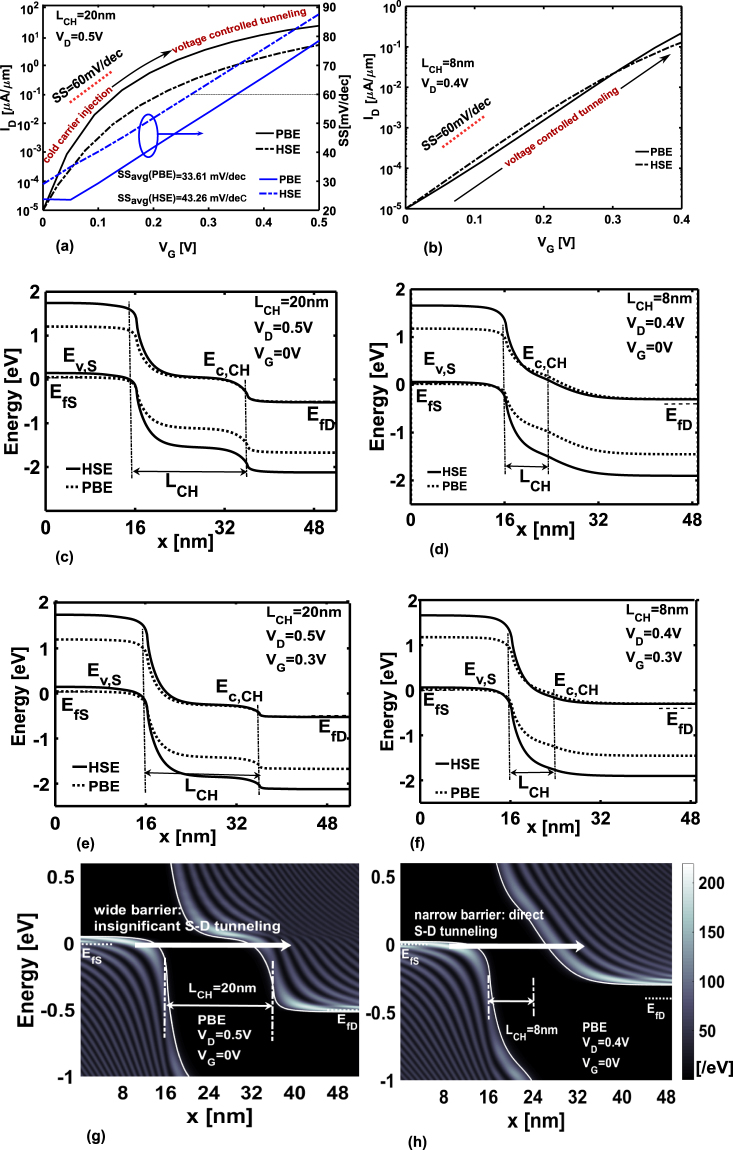
Transfer characteristics in monolayer GeSe-TFET for (**a**) *L*_CH_ = 20 nm and (**b**) *L*_CH_ = 8 nm with respect to both HSE and PBE methods. Energy band profile at *V*_G_ = 0 V for (**c**) *L*_CH_ = 20 nm and (**d**) *L*_CH_ = 8 nm and at *V*_G_ = 0.3 V for (**e**) *L*_CH_ = 20 nm and (**f**) *L*_CH_ = 8 nm. LDOS plots at OFF-state for (**g**) *L*_CH_ = 20 nm and (**h**) *L*_CH_ = 8 nm where S and D stand for source and drain respectively. In (**a**) the point SS is plotted on the right y-axis. Average SS is calculated by considering the threshold voltage at *V*_D_/2^11^.

The ON-current, *I*_ON_ obtained from self-consistent calculations are listed in Table 2, for all the devices. Careful examination of the vaues in the Table reveal that the difference in magnitude of ON-current between HSE and PBE diminishes as the channel length is scaled down. In order to explain this phenomenon we consider two cases, *L*_CH_ = 20 nm at *V*_D_ = 0.5 V and *L*_CH _= 8 nm at *V*_D_ = 0.4 V, for which we plot the transfer characteristics in Fig. 4. The reason for such difference in transfer characteristics lies in the fact that, in longer channel length devices (*L*_CH_ ≥ 10 nm) the transport is dominated by “cold carrier injection” whereas in shorter length devices, it is controlled by “voltage controlled tunneling”^59^. As pointed out in^59^ and also what we find in Fig. 4(a), (b) is that, “voltage controlled tunneling” does not have significant effect on the lowering of SS below the Boltzmann limit and does not vary considerably with gate voltage.

To elaborate this further, we refer to Fig. 4(c)–(f) where we plot the energy-band diagrams for *L*_CH_ = 20 nm at *V*_D_ = 0.5 V and *L*_CH_ = 8 nm at *V*_D_ = 0.4 V for both OFF (*V*_G_ = 0 V) and ON-states (*V*_G_ = 0.3 V). We also show the local density of states (LDOS) plots for the above-mentioned cases at  *V*_G_ = 0 V with respect to PBE. It is worth mentioning that similar behavior of LDOS is also observed in HSE (see Supplementary Information). If we examine the LDOS plot in Fig. 4(g) for longer channel TFET at OFF-state, we find that the electrons adjacent to source Fermi level, *i*.*e.* the region where the current spectrum peaks (see Supplementary Information) see a higher barrier while crossing over to the channel-drain junction in order to fill the empty states. But as *V*_G_ increases there is a significant opening up of the tunneling window. This can be seen from the potential energy diagram in Fig. 4(c),(e) showing a well defined crossing over of *E*_v,S_ and *E*_c,CH_ from OFF to ON-state. As a result electrons from the source see a thinner tunneling barrier and higher availability of empty states in the channel. The crossing over of the band edges is resposible for the “low-pass filtering” of the high energy tails of the Fermi distribution function^60^ of the source side. As a result, in the low *V*_G_ regime, transport in long channel TFET is determined by the “cold carrier injection”^59^ which entails the ${\rm{S}}{\rm{S}}\approx ln(10)\,\frac{{\rm{\Delta }}\varphi }{q}$^59,60^, where Δ*ϕ* = *E*_v,S_ − *E*_c,CH_. From our calculations it is found that, Δ*ϕ* ≈ 0.01 eV in HSE TFET and 0.001 eV in PBE TFET. Due to the linear dependence of SS on Δ*ϕ*^60^, the PBE TFET shows a larger regime of SS ≤ 60 mV/dec with respect to gate bias (see Fig. 4(a)) and therefore higher drain current compared to HSE TFET in this low *V*_G_ regime. As *V*_G_ increases *i*.*e.* in the post-subthreshold regime, value of Δ*ϕ* increases and the transport mechanism shifts to “voltage controlled tunneling”^59^. The SS in both HSE and PBE does not change significantly with gate voltage in this high *V*_G_ regime (see Fig. 4(a)). However, since the BTBT current is proportional to tunneling probability under Wentzel–Kramers–Brillouin approximation (*T*_WKB_), where *T*_WKB_ = expξ_*I*_/*F*^59^, with *F* corresponding to the electric field at the source-channel junction and ${\xi }_{I}=4\sqrt{2{m}_{r}^{\ast }}{E}_{G}^{3/2}/3(q\hslash )$, where *q* is the electronic charge, ${m}_{r}^{\ast }$ is the reduced tunneling mass and *ħ* = *h*/(2*π*), *h* being the Planck’s constant, therefore PBE TFET due to higher electric field *F* resulting from its low band gap value, gives rise to significantly higher ON-state current.

In case of shorter channel TFETs (*L*_CH_ ≤ 10 nm), the electrons in the source see a smaller and thinner tunneling barrier in the OFF-state and are able to directly tunnel through to the channel-drain junction to fill up the empty states (see LDOS plot in Fig. 4(h)). The higher probabilty of direct source to drain tunneling in shorter channel TFET in low *V*_G_ regime, ultimately renders the minor (with respect to long channel TFET) crossing over of *E*_c,CH_ and *E*_v,S_ (see Fig. 4(d),(f)) from OFF to ON-state, ineffective. This eventually results in over-passing the “filtering effect of the source Fermi function” and forces the device to be in the “voltage controlled tunneling” regime. Mathematically, the SS is now determined as^59,60^ $\approx \frac{{ln}(10){({E}_{G}+{\rm{\Delta }}\varphi )}^{2}}{{q}^{2}{\rm{\Lambda }}{\xi }_{I}}$, where Λ = *W*_*D*_ + *λ*, with *W*_*D*_ and *λ* corresponding to the depletion width at the source-channel junction and geometric screening length respectively. In the best case, the value of SS is very close to the thermal limit^59^. In our case, SS turns out to be >60 mV/dec (95.2 mV/dec for PBE and 83.6 mV/dec for HSE). For the chosen gate voltage regime, the values of SS show insignificant difference between HSE and PBE TFETs, and remain almost constant over the range of *V*_G_ (see Fig. 4(b)). This eventually results in minimal change of *I*_D_ between HSE and PBE TFETs.

## Discussion

**Figure 5:**
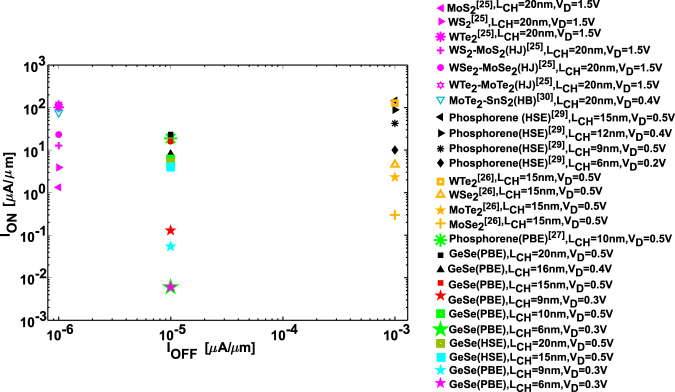
Comparison of *I*_ON_ with other 2D material based TFETs. HJ and HB correspond to hetero-junction and hetero-bilayer respectively whereas PBE and HSE correspond to the functional of the DFT–PAW method used to calculate the electronic structure of the material.

First we present a comparison of the ON-current obtained in our device with respect to other reported 2D material based TFETs. It is worth noting that achieving hetero-bilayer materials with direct band gap is difficult^61,62^. However, theoretical calculations^30^ are conducted under ballistic assumption. From Fig. 5, we find that irrespective of the scaling rule, GeSe-TFETs with *L*_CH_ ≥10 nm show comparable *I*_ON_ as other reported TMD and Phosphorene TFETs.

Next we discuss about the dynamic performance metrics, expressed in terms of intrinsic switching delay (*τ*), and power delay product (PDP). The intrinsic switching delay is computed following the method explained in^63^ as *τ* = (*Q*_ON_ − *Q*_OFF_)/*I*_ON_, where *Q*_ON_ and *Q*_OFF_ are the overall charges in the device at ON and OFF-state, respectively. The PDP is calculated as *V*_D_(*Q*_ON_ − *Q*_OFF_)^64^.

**Figure 6:**
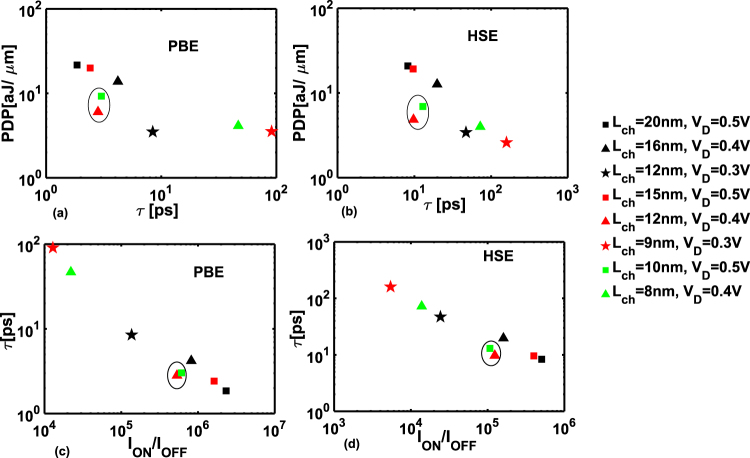
PDP versus intrinsic switching delay graph for (**a**) PBE and (**b**) HSE. Intrinsic switching delay versus *I*_ON_/*I*_OFF_ for (**a**) PBE and (**b**) HSE. The circled nodes are the most optimized in performance.

From the PDP-delay curve in Fig. 6(a) and (b) it can be seen that for a particular scaling rule the PDP decreases with the scaling of *L*_CH_ and *V*_D_ (go vertically down in Table 2). This is due to the decrease in values of *Q*_ON_ and *Q*_OFF_ as *L*_CH_ and *V*_D_ are reduced. However, *τ* increases due to the degradation of ON-current. If we go horizontally in Table 2, *i*.*e*. *L*_CH_ is scaled keeping *V*_D_ constant, the PDP decreases due to decrease in the charge content in smaller devices, but *τ* fluctuates between nodes depending on the value of *I*_ON_ and the ratio (*Q*_ON_ − *Q*_OFF_)/*I*_ON_. The best performant devices should lie in lower left corner of the graph. We find two devices in that region, namely *L*_CH_ = 10 nm at *V*_D_ = 0.5 V, *i*.*e*. the first node in the column of scaling rule = 20 nm/V and *L*_CH_ = 12 nm at *V*_D_ = 0.4 V *i*.*e*. the second node in the column of scaling rule = 30 nm/V. Interestingly, these two nodes represent the optimally designed GeSe-TFETs with respect to PDP-delay for both PBE and HSE calculations.

Next we analyze the *τ* − *I*_ON_/*I*_OFF_ graph in Fig. 6(c) and (d). Here the best performant devices should lie in the lower right corner. For a particular scaling rule *I*_ON_/*I*_OFF_ ratio decreases as a function of *L*_CH_ and *V*_D_ and *τ* increases. The degradation in values of *τ* becomes prominent as devices are scaled below *L*_CH_ = 12 nm due to the rapid decrease of *I*_ON_ compared to (*Q*_ON_ − *Q*_OFF_). Even so, we still find the two nodes pointed out previously, lying in the lower right corner of the graph demonstrating *I*_ON_/*I*_OFF_ > 10^4^ and *τ* = 3 ps. Moreover, in all of the plots we find that the devices considered under scaling rule 30 nm/V perform better in terms of switching speed and energy consumption.

In conclusion, monolayer GeSe-TFETs show appreciable performance with respect to *I*_ON_ − *I*_OFF_ ratio while scaling down to 0.3 V supply voltage with 9 nm channel and to 8 nm channel with a supply voltage of 0.4 V. It is also worth noting that, PBE TFETs always outperform HSE TFETs in case of *I*_ON_ requirements due to its higher tunneling probability arising from lower band gap value. However, with respect to the dynamic metrics both HSE and PBE TFETs exhibit a common trend in terms of PDP-delay and delay-*I*_ON_/*I*_OFF_, with certain nodes dominating in switching speed and dynamic power dissipation. Apart from these, all the devices show comparable ON-current values with other 2D material based homojunction-TFETs. Therefore from our studies it can be predicted that monolayer GeSe can be seen as a potential candidate for 2D material based TFET in the future due to its interesting electronic properties and better air stability.

## Methods

DFT calculations are carried out using generalized gradient approximation (GGA) as implemented in the code VASP^65–67^ with PAW^68^ method using the PBE exchange-correlation functional. Also, the HSE hybrid functional is used to determine electronic properties. A plane wave cutoff energy of 545 eV is used and a Γ-centered 8 × 8 × 1 (in X, Y and Z directions) k-mesh is found to be suitable to sample the Brillouin zone. Electronic convergence is achieved when the difference in energy of successive electronic steps becomes less than 10^−4^ eV, whereas the structural geometry is optimized until the maximum force on every atom falls below 0.01 eV/Å. A large vacuum space of more than 20 Å in the direction of Z is applied to avoid any interaction between successive layers.

For generating CBS, we perform DFT calculations using Atomistix Tool Kit (ATK)^69^, where the accuracy of the calculations largely depends on the selection of norm-conserving pseudopotentials and numerical LCAO basis sets. We employ the GGA as the exchange correlation in conjunction with the PBE functional. We use “SG15” norm-conserving pseudopotentials with “Medium” basis sets. The optimized “SG15” provide smooth pseudopotentials with multiple projectors and non-linear core corrections^70,71^. For the purpose of obtaining the electronic structure of monolayer GeSe using “SG15” pseudopotentials, we set the k-points in the Monkhorst-Pack grid as 9 × 9 × 1(in X, Y and Z directions). The density mesh cutoff, in the numerical accuracy settings, is taken as 150 Hartree.

For simulating transport we conceive a two-band *k* ⋅ *p* hamiltonian^72,73^ which is given as

1 $$H({\bf{k}})\equiv [\begin{array}{cc}{a}_{c}{{k}_{x}}^{2}+{b}_{c}{{k}_{y}}^{2} & \gamma {k}_{x}\\ \gamma {k}_{x} & {a}_{v}{{k}_{x}}^{2}+{b}_{v}{{k}_{y}}^{2}\end{array}]\,,$$

where **k** = (*k*_*x*_, *k*_*y*_) is the in-plane wave vector. It is worth noting that since no other bands are present near the CBM and VBM in the energy window (−1 to 2 eV), therefore the two-band model is sufficient to capture transport in our device. The off-diagonal first order *k*_*x*_ terms in the hamiltonian are responsible for opening a band gap^72^. The fitting parameters used in calibration of the *k* ⋅ *p* model are listed in Table 3.

**Table 3:** Fitting parameters of *k* ⋅ *p* hamiltonian obtained by calibrating against DFT results.

Method	Fitting Parameters for *k* · *p* hamiltonian				
	*a*_*c*_(eV*Å*^2^)	*a*_*v*_(eV *Å*^2^)	*b*_*c*_(eV *Å*^2^)	*b*_*v*_(eV *Å*^2^)	*γ*(eV *Å*)
DFT-PAW PBE	4.581	3.19	9.453	8.594	5.28
DFT-PAW HSE	5.105	4.211	17.188	16.441	5.92

The electron and hole effective masses along the transport direction as calculated from the *k* ⋅ *p* model for PBE are 0.1317 *m*_0_ and 0.1384 *m*_0_ respectively whereas for HSE are 0.1401 *m*_0_ and 0.1449 *m*_0_ respectively. Next we model the transport by self-consistently solving the transport equations based on NEGF formalism with the Poisson’s equation. We assume the device to be invariant along the transverse direction *y* and impose Born-von Karman periodic boundary conditions. This enables us to parameterise the hamiltonian and the Green’s functions in terms of the transversal wave vector *k*_*y*_. The retarded Green’s function^74,75^ for each wave vector *k*_*y*_, is calculated as

2 $${G}^{r}({k}_{y},E)={[EI-H({k}_{y})-{{\rm{\Sigma }}}_{S}-{{\rm{\Sigma }}}_{D}]}^{-1}$$

where *E* is the energy, *H*(*k*_*y*_) is the hamiltonian matrix discretized along the transport direction *x*, *I* is the identity matrix and Σ_*S*_/Σ_*D*_ are self-energy matrices associated to the source/drain contacts. From the retarded Green’s function *G*^*r*^(*k*_*y*_, *E*), we eventually calculate the electron and hole Green’s functions: *G*^*n*^(*k*_*y*_, *E*) and *G*^*p*^(*k*_*y*_, *E*) and the current density from position *i* to *i* + 1 along the *x* direction as follows:

3 $$\begin{array}{l}{G}^{n}({k}_{y},E)={G}^{r}({k}_{y},E){{\rm{\Sigma }}}_{n}{G}^{r\dagger }({k}_{y},E)\\ {G}^{p}({k}_{y},E)={G}^{r}({k}_{y},E){{\rm{\Sigma }}}_{p}{G}^{r\dagger }({k}_{y},E)\\ {{J}^{n(p)}}_{i,i+1}=\sum _{{k}_{y}}\frac{2q}{\hslash S}{\int }_{-\infty }^{+\infty }\frac{dE}{2\pi }[{H}_{i,i+1}{{G}^{n(p)}}_{i+1,i}({k}_{y},E)-{H}_{i+1,i}{{G}^{n(p)}}_{i,i+1}({k}_{y},E)]\end{array}$$

where

4 $$\begin{array}{l}{{\rm{\Sigma }}}_{n}\equiv ({{\rm{\Sigma }}}_{S/D}-{{{\rm{\Sigma }}}_{S/D}}^{\dagger })\cdot {f}_{S/D}\\ {{\rm{\Sigma }}}_{p}\equiv ({{\rm{\Sigma }}}_{S/D}-{{{\rm{\Sigma }}}_{S/D}}^{\dagger })\cdot (1-{f}_{S/D})\end{array}$$

with † denoting the transpose conjugate and *f*_*S*/*D*_ denoting the Fermi-Dirac distribution function at source/drain. The 2D Poisson’s equation is solved in a cross-section in the *x*–*z* plane. The discretization is based on the finite difference method, enforcing Dirichlet boundary conditions at the metal gate electrodes and Neumann boundary conditions on the rest of the edges.

## Electronic supplementary material

Supplementary information
