# Supplementary material for: Scalability assessment of Group-IV mono-chalcogenide based tunnel FET
Source: Sci Rep. 2018 Apr 16;8:5993. doi: 10.1038/s41598-018-24209-1 (PMC5902579; doi:10.1038/s41598-018-24209-1)
Supplement: Supplementary file 1 — Supplementary information [file 41598_2018_24209_MOESM1_ESM.pdf]

Supplementary materials

Scalability assessment of Group-IV monochalcogenide based tunnel FET

Madhuchhanda Brahma[1, 2], Arnab Kabiraj[2], Dipankar Saha[2], Santanu Mahapatra[2]

1. Centre for Nano Science and Engineering, Indian Institute of Science, Bangalore, 560012, India
2. Nano-Scale Device Research Laboratory, Department of Electronic Systems Engineering, Indian Institute of Science, Bangalore, 560012, India

Table T1 Values of doping concentration and length of source drain extensions considered during self- consistent simulation

<table>
<tr><th rowspan="3">V_D (V)</th><th rowspan="3"></th><th colspan="6">Scaling Rule (nm/V)</th></tr>
<tr><th colspan="2">40</th><th colspan="2">30</th><th colspan="2">20</th></tr>
<tr><th>HSE</th><th>PBE</th><th>HSE</th><th>PBE</th><th>HSE</th><th>PBE</th></tr>
<tr><td rowspan="4">0.5</td><td>L_{CH} (nm)</td><td colspan="2">20</td><td colspan="2">15</td><td colspan="2">10</td></tr>
<tr><td>L_D (nm)</td><td colspan="4">16</td><td colspan="2">25</td></tr>
<tr><td>N_S (x10^{17}/m^2)</td><td>2</td><td>1</td><td>2</td><td colspan="3">1</td></tr>
<tr><td>N_D (x10^{17}/m^2)</td><td colspan="4">0.05</td><td colspan="2">0.01</td></tr>
<tr><td rowspan="4">0. 4</td><td>L_{CH} (nm)</td><td colspan="2">16</td><td colspan="2">12</td><td colspan="2">8</td></tr>
<tr><td>L_D (nm)</td><td colspan="2">16</td><td colspan="4">25</td></tr>
<tr><td>N_S (x10^{17}/m^2)</td><td>2</td><td colspan="4">1</td><td>0.6</td></tr>
<tr><td>N_D (x10^{17}/m^2)</td><td colspan="2">0.5</td><td colspan="4">0.01</td></tr>
<tr><td rowspan="4">0.3</td><td>L_{CH} (nm)</td><td colspan="2">12</td><td colspan="2">9</td><td colspan="2">6</td></tr>
<tr><td>L_D (nm)</td><td colspan="6">25</td></tr>
<tr><td>N_S (x10^{17}/m^2)</td><td>1.5</td><td colspan="3">1</td><td colspan="2">0.6</td></tr>
<tr><td>N_D (x10^{17}/m^2)</td><td colspan="6">0.01</td></tr>
</table>

From the Table T1 we find that as L_{CH} is decreased the drain doping N_D is reduced to ensure lower OFF-state leakage. Also, the source doping N_S is kept slightly higher in case of HSE TFETs with respect to PBE so as to achieve ON-state current comparable to PBE-TFET. This is due to the fact that higher N_S increases the tunneling window and hence the ON-current. At the same time, lowering N_D translates to a larger depletion width at the channel-drain junction which compelled to increase L_D so that the electric field converges to charge neutrality condition at the end of the drain extensions.

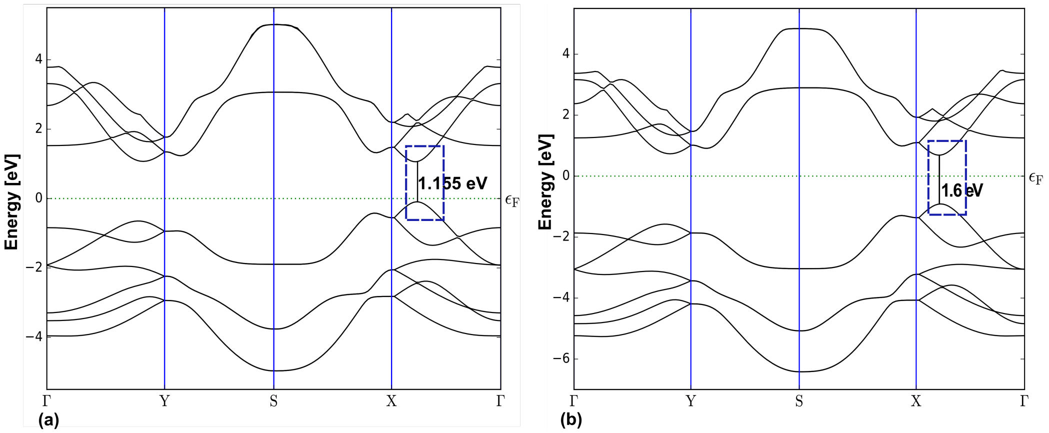

Figure S1 Band structure obtained from DFT-PAW by (a) PBE and (b) HSE methods. The rectangular dashed region denotes the energy window across which the k.p calibration is performed against DFT results.

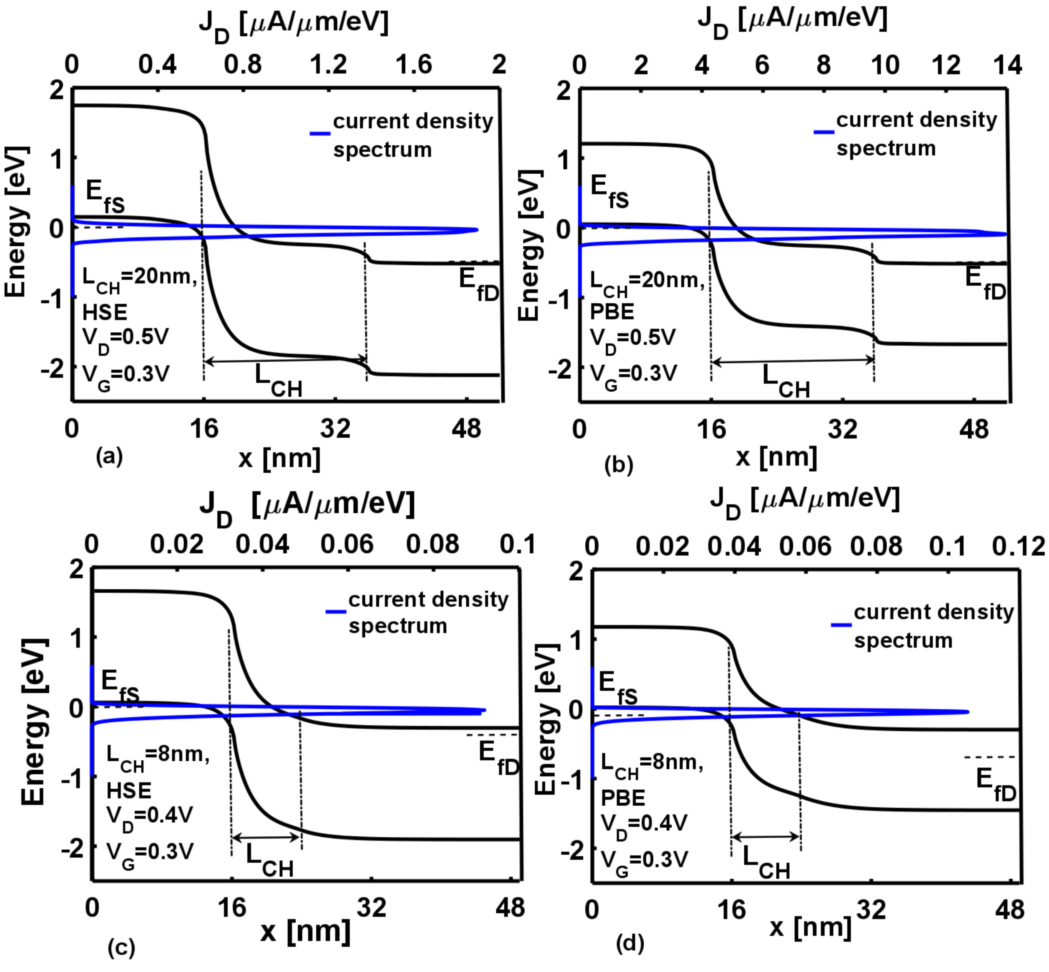

Figure S2 Energy resolved current density spectrum plots for L_{CH}=20nm, V_D=0.5V (a) HSE and (b) PBE TFETs and for L_{CH}=8nm, V_D=0.4V (c) HSE and (d) PBE TFETs all at same V_G=0.3V. In each case the current spectrum peaks around the source Fermi

level $E_{fS.}$ Also, difference between HSE and PBE current density values is higher in case of longer channel TFETs.

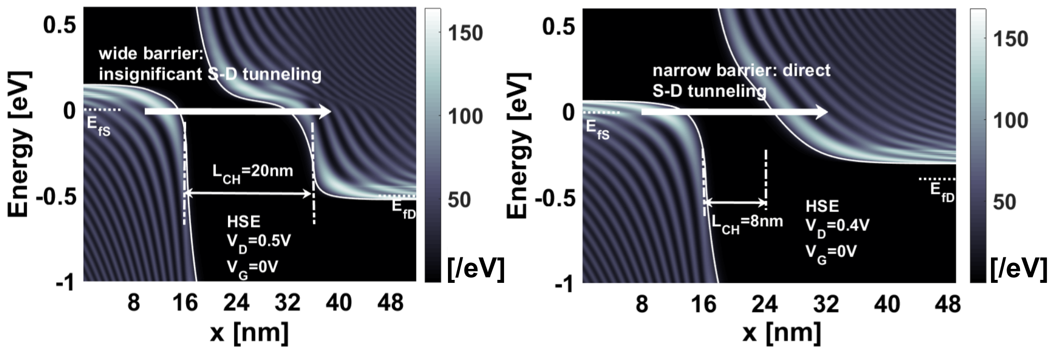

Figure S3 LDOS plots at V_G=0V for (a) L_{CH}=20nm, V_D=0.5V and (b) L_{CH}=8nm, V_D=0.3V for HSE TFET
